# User Profiles and Engagement in a Hypertension Self-Management App: Cross-Sectional Survey

**DOI:** 10.2196/83075

**Published:** 2026-02-11

**Authors:** Felix Muehlensiepen, Susann May, Frances Seifert, Eileen Wengemuth, Olen Johannsen, Martin Middeke, Martin Heinze, Pascal Petit, Nicolas Vuillerme, Sebastian Spethmann, Dunja Bruch

**Affiliations:** 1 Center for Health Services Research, Faculty of Health Sciences Brandenburg, Brandenburg Medical School, Rüdersdorf, Seebad 82/83, Ruedersdorf bei Berlin, 15562, Germany, 49 01636055860; 2 Department of Cardiology, Angiology and Intensive Care Medicine, Deutsches Herzzentrum der Charité, Berlin, Germany; 3 Charité – Universitätsmedizin Berlin, corporate member of Freie Universität Berlin and Humboldt-Universität zu Berlin, Berlin, Germany; 4 Université Grenoble Alpes, CNRS, Grenoble INP, LIG Sangria, Grenoble, France; 5 Hypertension Care UG, Ingolstadt, Germany; 6 Hypertension Center Munich, Munich, Germany; 7 Center for Mental Health, Brandenburg Medical School, Immanuel Klinik Rüdersdorf, Ruedersdorf bei Berlin, Germany; 8 Department of Cardiovascular Surgery, Heart Center Brandenburg, Faculty of Health Sciences, Brandenburg Medical School Theodor Fontane, Bernau, Germany

**Keywords:** digital health, mHealth, digital divide, hypertension, cardiology, chronic disease, questionnaire, health services research, user perspectives, equity, digital public health, mobile health

## Abstract

**Background:**

Mobile health (mHealth) technologies can improve hypertension self-management, yet real-world adoption remains limited and unequally distributed.

**Objective:**

This study aimed to characterize the profiles, usage patterns, and engagement of active users of a hypertension self-management app (*Hypertension.APP*) in Germany, with a focus on user engagement and potential digital divides.

**Methods:**

We conducted a cross-sectional online survey among adult users of *Hypertension.APP* in Germany between January and September 2023. An 88-item questionnaire assessed app usage patterns, perceived utility, integration into clinical care, sociodemographic and clinical data, and digital health literacy (eHealth Literacy Scale; scores 16‐40). Digital health literacy was categorized as low (16‐23.99), moderate (24‐31.99), or high (32-40). Descriptive statistics and univariable ordinal logistic regression were used to explore associations between sociodemographic and clinical variables and app usage frequency.

**Results:**

Of 254 respondents, the mean age was 53.6 years, and 54.3% (138/254) were male. A total of 44.5% (113/254) had a university or technical college degree, and 44.5% (113/254) reported a monthly net income higher than €2500 (US $2950). Most participants (224/254, 88.2%) reported access to at least two digital devices. Overall, 88.2% (224/254) had moderate or high digital health literacy (eHealth Literacy Scale ≥24). App engagement was high: 80.7% (205/254) reported using the app at least weekly, and 52.4% (133/254) reported using the app to prepare for medical visits. However, only 20.1% (51/254) reported that the app was formally integrated into their medical care, and 11.8% (30/254) indicated that medication had been adjusted based on app data. In univariable ordinal logistic regression analyses, higher education, longer duration of hypertension, and living in a small town (5000‐20,000 inhabitants) were associated with more frequent app use, whereas systolic blood pressure of 140 mm Hg or higher was associated with less frequent use. Digital health literacy was not clearly associated with app usage frequency among current users.

**Conclusions:**

Users of this hypertension self-management app were predominantly well-educated, digitally literate individuals with established hypertension, reinforcing concerns about a persistent digital divide. While app usability and engagement were high, formal clinical integration remained limited. Simply making an app available is insufficient; strategies to promote equitable access, strengthen clinical integration, and support patients with lower digital health literacy are needed for mHealth to contribute effectively to hypertension management.

## Introduction

Cardiovascular diseases remain the leading cause of death worldwide, despite the availability of evidence-based strategies for prevention and treatment [1]. Arterial hypertension—affecting more than 1 billion people globally—is a major modifiable risk factor [2]. In addition to genetic predisposition, the development and progression of high blood pressure are strongly influenced by individual lifestyle factors, health literacy, and behaviors [3], as well as by structural determinants such as access to and quality of medical care [4].

In recent years, digital health technologies have gained increasing relevance in the prevention and management of chronic conditions, including hypertension [5]. Mobile health (mHealth) applications, wearable devices, and other digital tools offer new opportunities for individuals to monitor their health, adopt healthier behaviors, and take a more active role in managing their condition. These tools also provide health professionals with novel ways to support daily care routines and improve treatment adherence [6].

Multiple studies have shown that mHealth interventions can contribute to better blood pressure control [7]. Most recently, Prendergast et al [8] demonstrated in a landmark single-center randomized clinical trial that an emergency department–based education and mHealth empowerment intervention for patients with hypertension led to significantly greater reductions in systolic blood pressure (SBP) at 6 months compared with standard discharge care.

However, findings from controlled trials may not translate to routine practice. Evidence from other areas of digital health suggests that adherence to mHealth interventions in everyday practice tends to be low [9-11]. Common barriers include limited awareness of mHealth benefits among both patients and health care professionals [12-14], insufficient reimbursement structures [15], and difficulties integrating digital tools into existing care pathways [16]. While these challenges have been widely discussed from the perspective of health systems and providers, less is known about the individuals who actively use such tools. Gaining insight into who uses hypertension apps, how they are used, and what users expect from them can support the development of more effective and user-centered digital health solutions.

In a preceding qualitative study [17], we explored patient users’ perspectives on a hypertension self-management app (*Hypertension.APP*), focusing on perceived benefits, potential risks, and the app’s role in everyday care. Patients valued features such as continuous monitoring and personalized feedback but reported limited integration into formal health care and expressed concerns about data privacy. Moreover, the findings suggested that users tended to be digitally literate, self-effective, and relatively well-educated. These qualitative insights provided important initial evidence but were based on a small sample (n=20). The present study aims to build on these findings using a quantitative approach to provide a broader and more generalizable understanding of app users in hypertension care.

The objective of this cross-sectional study was to investigate the profiles, usage patterns, and engagement of active users of a hypertension self-management app. Using a standardized survey, we examined user demographics, usage patterns, usability, and clinical integration of *Hypertension.APP*.

## Methods

### Study Design

This study was conducted as a cross-sectional study. It examines how users of *Hypertension.APP* evaluate their experiences and the app’s perceived impact on their hypertension care. The data provide insights into user attitudes, app engagement, and the role of digital health technologies in hypertension management.

This study is part of the Digital Preventive Measures for Arterial Hypertension project [18], which examines the structural and individual factors of patients and stakeholders in the use of digital preventive measures among patients with arterial hypertension in Germany.

### Setting

The study was conducted via online recruitment, targeting adult users of *Hypertension.APP* across Germany. Recruitment and data collection were carried out between January 1 and September 30, 2023. Users were approached within their real-world use context of the app. Potential participants were invited via 3 digital channels: (1) in-app notifications within *Hypertension.APP*, (2) email invitations sent to registered users, and (3) social media posts on channels maintained by the app provider and project partners. All invitations briefly described the purpose of the study, stated the eligibility criteria, and provided a link to the online questionnaire.

### Participants

Eligibility criteria included individuals aged 18 years or older who had used the app for at least 1 month. Exclusion criteria included individuals without access to a smartphone or tablet and those unable to provide informed consent. After clicking on the survey link, interested individuals were first presented with an online information sheet and an electronic informed consent form. Only participants who provided consent were forwarded to the actual questionnaire. Participation was voluntary and anonymous, and respondents could skip individual questions if they did not wish to answer them.

### Sample Size

The planned sample size was set at N=200, based on an a priori power analysis (G*Power) for the chi-square test with the following assumptions: medium effect (w=0.2), *α*=.05, power (1-β)=.80, *df*=1 (required sample size n=197). Due to the high willingness of app users to participate, the sample size was exceeded.

### mHealth App: *Hypertension.APP*

Based on expert recommendations (Prof Dr Martin Middeke and Prof Dr med Miklós Illyés), *Hypertension.APP* was selected as an example and examined through a standardized user survey. *Hypertension.APP* was developed and is maintained by Hypertension Care UG (Ingolstadt) [19]. The application had been downloaded in app stores nearly 11,000 times as of July 2025. It includes a diary function to document blood pressure data, a personalized guide that provides information based on the user’s health profile, an export function to generate reports for medical consultation, a reminder function, and interactive exercises. The app is freely accessible in various app stores. There is also a purchasable premium version that includes additional features.

Two authors (OJ and MM) are the founders and/or chief executive officers of Hypertension Care UG, the company that developed *Hypertension.APP*, the app evaluated in this study. To minimize potential conflicts of interest, their role in the research process was restricted. The academic research team (FM, SM, DB, PP, NV, SS) independently developed the questionnaire, conducted data collection, managed the dataset, and performed all statistical analyses. The app developers did not have access to raw data and were not involved in data analysis or interpretation. All authors contributed to manuscript drafting, but final decisions on analysis and interpretation were made by the independent academic team.

### Questionnaire

The development of the 88-item questionnaire followed a structured, multistep process. First, an initial item pool was generated based on preliminary findings from our preceding qualitative interview study on hypertension app use [17], existing literature on mHealth adoption, and theoretical frameworks related to self-management and digital health literacy. To ensure content validity, we integrated established measurement instruments, including the German version of the eHealth Literacy Scale (eHEALS) [20] and system usability scale (SUS) [21], and supplemented these with newly developed items capturing app-specific use cases, clinical integration, and user expectations. Second, the research team (FM, SM, EW, DB) iteratively refined item wording and domain structure to improve face validity, comprehensiveness, and consistency. This process included expert review by clinicians and digital health researchers with experience in hypertension management and questionnaire development (OJ, MM, SS). Third, the questionnaire was pilot-tested with a sample of 10 adult hypertension app users to evaluate clarity, item relevance, and response burden. Feedback from the pilot phase resulted in minor adjustments to wording, response options, and the sequence of questions to enhance comprehensibility and usability.

The final instrument (German version: Multimedia Appendix 1; English version: Multimedia Appendix 2) covered multiple domains: app usage patterns, integration into medical care, perceived utility of specific app features, usability (SUS), digital health literacy (eHEALS), clinical characteristics, health behaviors, and sociodemographic information.

### Variables

Primary variables included user demographics, eHealth literacy, app engagement patterns, and perceptions of app utility. eHealth literacy was assessed using the German version of the eHEALS scale [20] and categorized into “Low” (16‐23.99), “Moderate” (24‐31.99), and “High” (32–40) levels. These thresholds follow the categorization approach proposed by Monkman et al [22], who examined conceptual distinctions within the scale and recommended these score ranges to differentiate levels of digital health literacy. App utility was measured using the German version of the SUS [21]. App engagement metrics, such as frequency and duration of use, were assessed alongside subjective evaluations of the app’s role in hypertension care. Sociodemographic data, including age, gender, education, and place of residence, were also collected.

### Data Sources and Measurement

All questionnaire data were self-reported, including blood pressure values. Participants provided their most recent blood pressure measurement, and no standardized measurement protocol was imposed. Participants were instructed to report values documented in *Hypertension.APP* or measured through their usual home monitoring routines. Accordingly, systolic and diastolic values reflect self-reported blood pressure at or near the time of survey completion rather than standardized study measurements.

### Bias

Efforts to mitigate bias included the use of validated instruments, such as eHEALS scale [20] and SUS [22], and standardized survey procedures. Missing data were handled by including only observations with less than 20% missing values.

### Statistical Methods

Data analysis was performed using R statistical software (version 2023.06.2; R Core Team, 2023). Descriptive statistics were used to summarize participant characteristics, app usage patterns, and SUS scores. Subgroup analyses were conducted for app usage frequency to explore variations by demographic factors (age, gender, place of residence, and education level). SUS scores were reported descriptively for the overall sample without subgroup analyses. To examine whether sociodemographic variables and digital health literacy (eHEALS) predicted app usage frequency, a univariable ordinal logistic regression analysis was performed using the MASS package [23].

### Ethical Considerations

The study was approved by the Ethics Committee of Brandenburg Medical School Theodor Fontane (E-02‐20220620). The study was registered as part of the Digital Preventive Measures for Arterial Hypertension project at the German Clinical Trials Register (DRKS00029761). Primary data collection was conducted in compliance with the current data protection regulations of the General Data Protection Regulation and the Declaration of Helsinki. Participants provided informed consent before being forwarded the actual questionnaire. Data collection was anonymous, and no conclusions can be drawn about individuals in the presentation of results. Participants received €15.00 (US $17.53) as an incentive and compensation for participation. The anonymity of the survey data during incentivization was ensured by using a separate database and individual passwords, with the two datasets stored separately. Personal data used for incentivization were deleted. There was no formal affiliation or relationship between the hypertension app examined in this study and the overarching research project. The app was developed and operated independently, and the collection and analysis of usage data were conducted solely for scientific purposes within the scope of this study.

## Results

### Demographic Characteristics

A total of 254 individuals completed the survey. The mean age was 53.6 (SD 13.9) years, ranging from 18 to 83 years. The majority of participants identified as male (139/254, 54.7%) or female (109/254, 42.9%), with a small proportion identifying as nonbinary (2/254, 0.8%). Most respondents reported having completed secondary or higher education, with 113/254 (44.5%) holding a university or technical college degree. Regarding monthly net income, 113/254 (44.5%) reported earnings above €2500 (US $3000), while 36/254 (14.2%) earned less than €1500 (US $1800) per month. Income was not specified by 49/254 (19.3%) of participants.

Participants were geographically diverse. Over two-fifths (105/254, 41.3%) reported living in large cities (>100,000 inhabitants), while 38/254 (15%) resided in rural areas (<5000 inhabitants). Digital health literacy was measured using the eHEALS scale. Among participants, 183/254 (72%) demonstrated moderate or higher levels of digital health literacy. Specifically, 20/254 (8%) of users fell into the “Low” category, 163/254 (64%) were in the “Moderate” category, and 71/254 (28%) were classified as having “High” eHealth literacy. Overall, 216/254 (85%) of participants reported knowing how to find health-related information online, but only 90/254 (35.4%) felt confident making health decisions based on online information.

An overview of sociodemographic characteristics is provided in Table 1.

**Table 1.** Sociodemographic characteristics of adult users of *Hypertension.APP* participating in a cross-sectional online survey in Germany (N=254; January–September 2023). The table includes age, sex, education, income, place of residence, and digital health literacy.

Characteristic	Values, n (%)	
Sex		
Male	139 (54.7)	
Female	109 (42.9)	
Nonbinary	2 (0.8)	
Missing	4 (1.6)	
Education level		
No degree	2 (0.8)	
Secondary school certificate	27 (10.6)	
Intermediate school diploma	42 (16.5)	
High school diploma	56 (22)	
University/technical college degree	113 (44.5)	
Other degree	7 (2.8)	
Missing	7 (2.8)	
Income (monthly net income)		
<€1000 (US $1200)	16 (6.3)	
€1000‐1500 (US $1200-1800)	20 (7.9)	
€1500‐2500 (US $1800-3000)	51 (20.1)	
>€2500 (US $3000)	113 (44.5)	
Not specified	49 (19.3)	
Missing	5 (2)	
Size of place of residence		
Rural (<5000 inhabitants)	38 (15)	
Small town (5000-20,000)	64 (25.2)	
Medium-sized town (20,000-100,000)	42 (16.5)	
Large city (>100,000)	105 (41.3)	
Missing	5 (2)	
Digital health literacy (eHEALS^a^)		
Low (16‐23.99)	26 (10.2)	
Moderate (24‐31.99)	98 (38.6)	
High (32-40)	126 (49.6)	
Missing	4 (1.6)	
Availability of digital technologies		
Smartphone	244 (96.1)	
Computer or laptop	223 (87.8)	
Tablet	173 (68.1)	
Landline phone	164 (64.6)	
Smartwatch/fitness tracker	124 (48.8)	

a eHEALS: eHealth literacy scale.

### Clinical Characteristics

Table 2 summarizes the clinical profile of participants. The majority of respondents reported SBP values between 120 and 139 mm Hg (158/254, 62.2%), and 24.8% (63/254) reported values at or above 140 mm Hg. Diastolic blood pressure was most frequently reported between 80 and 99 mm Hg (185/254, 72.8%), with 24.4% (62/254) indicating levels of 100 mm Hg or higher. Most participants (192/254, 75.6%) were currently taking antihypertensive medication. Regarding hypertension history, 21.3% (54/254) reported a diagnosis within the past year, while 35% (89/254) had been diagnosed more than 5 years ago. Notably, 6.3% (16/254) indicated that they had never been diagnosed with high blood pressure or no longer had the condition. In terms of smoking behavior, 9.1% (23/254) of respondents were current smokers, 32.3% (82/254) were former smokers, and 57.9% (147/254) had never smoked regularly.

**Table 2.** Clinical characteristics of adult users of *Hypertension.APP* in Germany, collected as part of a cross-sectional online survey (January–September 2023). Variables include blood pressure categories, comorbidities, medication use, duration of hypertension, and smoking status.

Characteristic	Values, n (%)	
Systolic blood pressure		
<120 mm Hg	31 (12.2)	
120‐129 mm Hg	67 (26.4)	
130‐139 mm Hg	91 (35.8)	
140‐159 mm Hg	54 (21.3)	
160‐179 mm Hg	9 (3.5)	
>180 mm Hg	0 (0)	
Missing	2 (0.8)	
Diastolic blood pressure		
<80 mm Hg	3 (1.2)	
80‐84 mm Hg	68 (26.8)	
85‐89 mm Hg	57 (22.4)	
90‐99 mm Hg	60 (23.6)	
100‐109 mm Hg	56 (22)	
>110 mm Hg	6 (2.4)	
Missing	4 (1.6)	
Antihypertensive medication use		
Yes	192 (75.6)	
No	60 (23.6)	
Missing	2 (0.8)	
Time since hypertension diagnosis		
<1 y	54 (21.3)	
1‐5 y	93 (36.6)	
5‐10 y	29 (11.4)	
>10 y	60 (23.6)	
Never diagnosed or no longer hypertensive	16 (6.3)	
Missing	2 (0.8)	
Smoking status		
Current smoker	23 (9.1)	
Former smoker	82 (32.3)	
Never smoked regularly	147 (57.9)	
Missing	2 (0.8)	
Comorbidities		
Elevated cholesterol levels	69 (27.2)	
Other chronic or serious medical conditions	48 (18.9)	
Physical disability	28 (11)	
History of heart attack or stroke	22 (8.66)	
History of cardiac or vascular surgery	12 (4.72)	
Diagnosed with diabetes mellitus	8 (3.15)	
Diagnosed with chronic kidney disease	6 (2.36)	
Means of transportation to go to the doctor		
Car	180 (70.9)	
Public transportation	116 (45.7)	
Bike	109 (42.9)	
Other means of transport	32 (12.6)	

The mean reported travel time to the doctor was 18.4 (SD 18.8) minutes, ranging from 0 to 159 minutes.

### App Usage and User Experience

**Figure 1.**
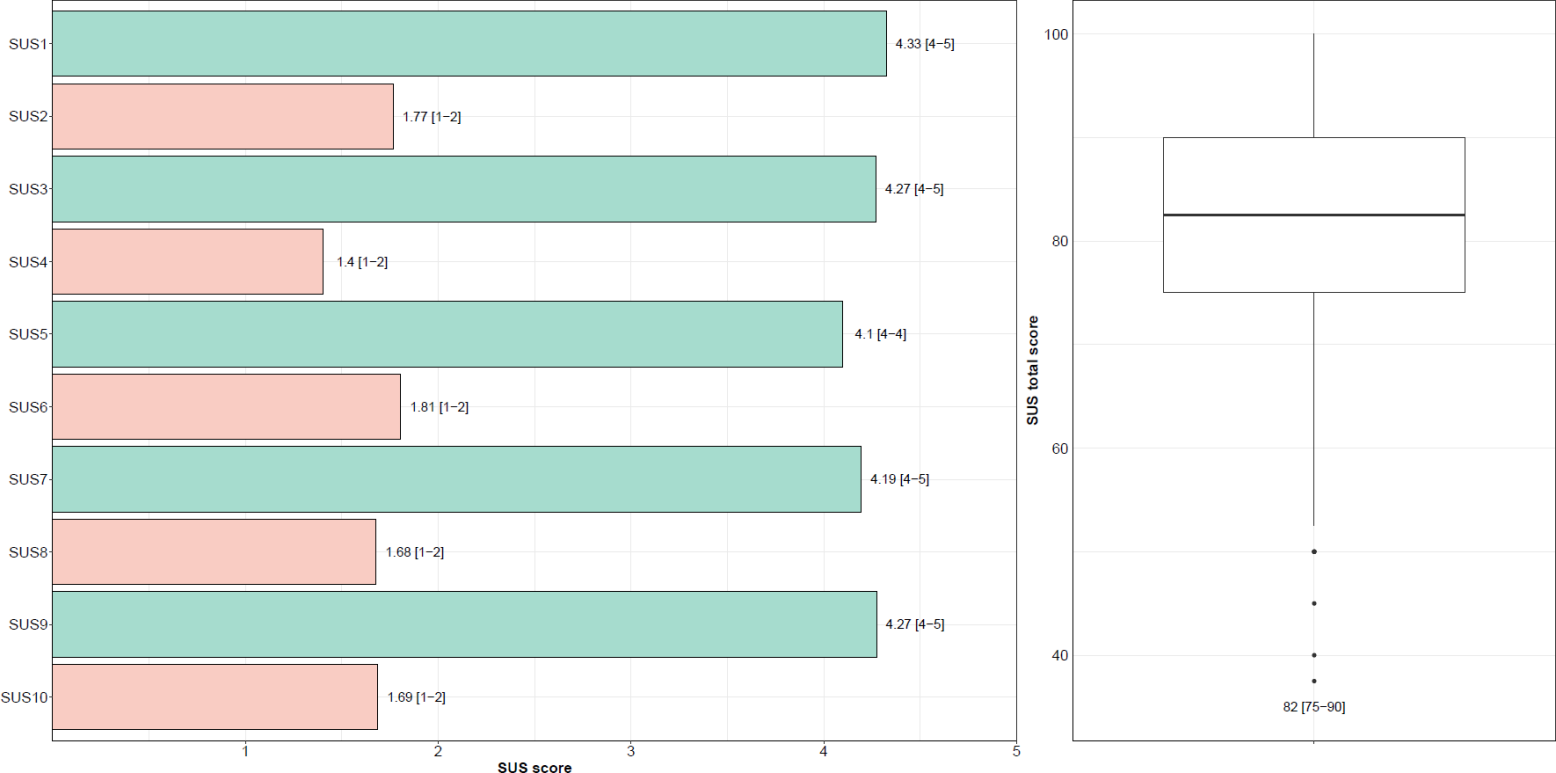
System usability scale (SUS) ratings of *Hypertension.App* among adult users in Germany participating in a cross-sectional survey (January-September 2023). The figure displays item-level responses (left) and the distribution of total SUS scores (right). Values represent mean (range)

Figure 1 summarizes the results of the 10-item SUS for all 254 participants. *Hypertension.APP* achieved a mean SUS score of 82 (SD 11.98; range 38‐100), indicating “excellent” usability according to established benchmarks.

Most respondents (239/254, 94.1%) reported actively using the app at the time of the survey; 52.4% (133/254) used it daily and 80.7% (205/254) at least once per week. Table 3 provides an overview of motivations for app use, specific use cases, and the extent to which the app was integrated into medical care.

**Table 3.** App usage patterns, reasons for use, integration into medical care, and perceived usefulness among adult users of *Hypertension.APP* in Germany, based on a cross-sectional online survey conducted between January and September 2023 (N=254).

Characteristic (multiple responses possible/top 5)	Values, n (%)	
Source of app awareness		
Independently searched for blood pressure apps online or in the app store	112 (44.1)	
Recommendation by a doctor	45 (17.7)	
Advertisement in print media	30 (11.8)	
Recommendation from friends/family	27 (10.6)	
Online advertisement	20 (7.9)	
Learned about the app in another way	31 (12.2)	
Reasons for using the app (user motivations)		
To document blood pressure	220 (86.6)	
To get information about high blood pressure	107 (42.1)	
To do something for one’s health	76 (29.9)	
Own curiosity	75 (29.5)	
To try deep breathing function	34 (13.4)	
App use cases (features actually used)		
Documentation blood pressure values	242 (95.3)	
Personalized health information suggestions	169 (66.5)	
Diary function	102 (40.2)	
General health information	96 (37.8)	
To create reports for doctor's visits	86 (33.9)	
App integrated into medical treatment		
Using blood pressure records to prepare for doctor’s visits	133 (52.4)	
App not yet integrated, but planned for future use	76 (29.9)	
App not currently integrated into care	51 (20.1)	
Medication adjusted by physician based on patient-reported entries	30 (11.8)	
Discussing advice or health information with physician	14 (5.5)	
Best feature of the app		
Blood pressure documentation	230 (90.6)	
Direct feedback on blood pressure values	138 (54.3)	
Preparation of reports for doctor's visit	111 (43.7)	
Personalized health information	98 (38.6)	
Diary entries	77 (30.3)	

To explore potential predictors of usage frequency, we conducted univariable ordinal logistic regression analyses (full results are provided in Multimedia Appendix 3). Of the 10 sociodemographic and clinical variables examined, 4 were associated with app usage frequency. Having a SBP of 140 mm Hg or higher was associated with less frequent app use, whereas living in a small town (5000‐20,000 inhabitants), having received an initial hypertension diagnosis more than 1 year ago, and having a higher level of education were associated with more frequent app use.

Participants reported using the app for multiple purposes. The most common use cases were documentation of blood pressure values (242/254, 95.3%), accessing personalized health information (169/254, 66.5%), and using the diary function (102/254, 40.2%). More than half of participants (133/254, 52.4%) indicated that they used the app to prepare for doctor’s visits. However, only a minority reported that the app had been formally integrated into their medical care (51/254, 20.1%) or that their physicians had adjusted medications based on app data (30/254, 11.8%). When asked about the app’s most helpful features, participants most frequently cited blood pressure documentation (230/254, 90.6%), direct feedback on blood pressure values (138/254, 54.3%), and preparation of reports for medical visits (111/254, 43.7%).

Awareness of the app was most commonly gained through self-initiated searches in the app store or online (112/254, 44.1%), followed by recommendations from health care professionals (45/254, 17.7%), advertisements in print media (30/254, 11.8%), and recommendations from friends or family (27/254, 10.6%).

## Discussion

### Overview

This cross-sectional survey provides insight into the profiles, usage patterns, and engagement of active users of a mobile app in hypertension care. Most users were digitally literate, middle-aged or older adults with higher education levels and above-average income. While app utility ratings and engagement were generally high, especially for documentation and preparation of doctor visits, formal integration of the app into clinical care remained limited.

### Who Uses the App? The Usual Suspects

Taken together, our data underscore a persistent digital divide [24]. The user base of the app largely reflects the “usual suspects” of digital health adoption: individuals with higher levels of education and income, often residing in urban or semiurban areas, and possessing moderate to high digital health literacy. These users are typically proactive in seeking out digital tools, show high engagement, and report favorable usability experiences (SUS). In contrast, individuals with lower digital competencies or limited access to technology were underrepresented, even though such groups often bear a disproportionate hypertension burden and face greater challenges in self-management [25].

This pattern aligns closely with prior research. A 2025 Dutch study demonstrated that people with a low socioeconomic position had significantly less access to and use of eHealth, whereas individuals with a higher socioeconomic position showed substantially greater utilization [26]. One key explanatory factor is eHealth literacy: highly educated individuals generally have stronger eHealth literacy and comfort with technology [26], which lowers barriers to using health apps. Conversely, individuals from disadvantaged backgrounds often face multiple obstacles, including limited skills, fewer resources, or lower confidence, and are therefore more likely to adopt new digital health tools later—or not at all [26].

Interestingly, digital health literacy was not associated with app usage frequency in our sample. At first glance, this appears to contradict previous research. However, our sample consisted exclusively of active app users, which implies a pronounced self-selection process: individuals with lower digital health literacy are less likely to adopt such tools in the first place. Among those who already use an app, variability in digital literacy is typically reduced, and literacy may no longer influence usage intensity. Moreover, eHEALS measures perceived rather than actual digital skills, which may further attenuate associations. Taken together, these factors likely explain why digital health literacy did not emerge as a significant predictor of usage frequency in this study.

Consistent with our findings, prior surveys in the United States and Europe have similarly found that health app users are typically younger or middle-aged adults with higher income and education levels [27,28]. Thus, despite the widespread availability of mHealth apps, their use remains socially patterned and disproportionately concentrated among individuals with higher socioeconomic status and digital competencies, as noted by multiple researchers [24,29]. Our study reinforces this point: access alone is not uniform, and digital interventions in hypertension care may be inadvertently catering to an already empowered subset of patients.

Notably, participants with SBP of 140 mm Hg or higher used the app less frequently, suggesting that worse current control may correlate with lower digital engagement. Interestingly, a noteworthy proportion of participants reported diastolic blood pressure values above recommended thresholds. While we did not perform subgroup analyses exploring associations between location, sociodemographic features, or other predictors and blood pressure levels, this finding merits attention. Elevated diastolic readings may reflect limited hypertension control among specific subgroups, which could affect both engagement with self-monitoring tools and clinical outcomes. Future studies should explore whether such patterns are associated with geographic, educational, or behavioral factors and assess whether app usage may contribute to improved blood pressure control over time.

While causal inferences cannot be drawn, similar paradoxical patterns have been observed elsewhere. For example, a German survey reported lower health app use among individuals who perceived themselves at high diabetes risk [30]. Even after adjusting for demographics, high perceived risk was associated with lower app usage, suggesting that individuals with greater health concerns or disease burden might rely more on traditional care or face motivational barriers to using apps. These findings highlight the need for targeted strategies to better support and engage higher-risk patients in digital self-management.

### Implications for Research and Practice

Our results highlight an unmet need to make digital health technologies more inclusive and accessible. Currently, mHealth interventions often serve the “tech-savvy” but miss those with limited digital experience or lower health literacy, a pattern that can exacerbate health disparities [29]. To ensure these tools benefit a broader patient population, developers and health care providers should consider strategies to lower the barriers to entry for less digitally literate users. Recommended approaches include the following:

- Usability and language: Apps should use clear navigation and plain language. Short sentences improve comprehension, and intuitive icons and visuals can support users who struggle with text-heavy information. Furthermore, providing content and instructions in multiple languages is crucial in diverse populations [31].
- Low-threshold onboarding and education: It should be easy for new users to get started. This could involve in-app tutorials, community workshops, or integration with primary care so that health care staff can introduce and demonstrate the app. Such onboarding can build confidence for those who are less technologically experienced. Digital navigators [32] could serve as an important bridge between patients and technology and could play a key role in this process; however, they have yet to be widely adopted in routine care.
- Collaboration with health care providers (clinical integration): Stronger links between app data and primary care workflows would benefit both patients and clinicians. Integrating the app with electronic health records and enabling data sharing with physicians would enable routine use of these data for monitoring and decision-making during clinic visits.

From a health systems perspective, clinical integration is pivotal. Although patient engagement was high, incorporation of the app into routine care by providers was low—a disconnect commonly seen with mHealth tools. Experts argue that integrating patient-facing apps into standard clinical practice is essential for digital health to reach its full potential [33]. At present, most consumer health apps operate in isolation—patients may track their data, but it often stays on their phone and is not mandatorily reviewed by their clinician. In line with this, few physicians today formally recommend or prescribe health apps to their patients, often due to limited time and lack of information, reimbursement, or guidelines [12-16]. Addressing these barriers will require collaboration among developers, health care providers, and policy makers to ensure apps meet clinical standards, fit seamlessly into workflows, and address patient needs. Ultimately, stronger collaboration and integration are necessary for mHealth to move from a personal self-management aid to a recognized element of health care delivery.

### Limitations and Generalizability

To our knowledge, this is one of the first quantitative studies to systematically characterize real-world users of a hypertension self-management app in Germany. Prior work in this field has primarily relied on small qualitative samples or clinical trial settings, which often do not reflect everyday app usage. By combining detailed sociodemographic, clinical, and digital literacy data with real-world app engagement patterns, our study advances understanding of who uses hypertension apps and how these tools are integrated—or not integrated—into routine care. The finding that a digital divide persists even among active app users, as well as the inverse association between elevated SBP and frequency of app use, provides novel insights that have not been reported in previous hypertension mHealth research.

Yet, it is important to acknowledge the study’s limitations. First, our data come from a cross-sectional survey based on self-reported app use, behaviors, and blood pressure values. Self-reported data are susceptible to recall bias (participants might not remember their usage accurately) and social desirability bias (participants might overstate “good” behaviors, like app use, or underreport problems). In addition, blood pressure measurements were self-reported by users and not clinically validated, which may have introduced measurement inaccuracies or reporting bias. This is a common caveat in mHealth surveys, and while we took steps to anonymize responses, some bias cannot be ruled out.

Second, the sample is not representative of all individuals with hypertension or of all app users. Due to the self-selection recruitment process, the study inherently attracted participants who were receptive to research, which might also be associated with academic backgrounds. Furthermore, the convenience sample—recruited within *Hypertension.APP*—limits representativeness for all people with hypertension or all hypertension app users.

Third, Germany’s high-resource, digitally mature context may not reflect lower-resource settings. What we observed in an urban European context (high smartphone penetration, moderate-to-high digital literacy) might not directly translate to settings where access to smartphones, the internet, or health education is more limited.

A further limitation is that important sociodemographic factors known to influence mHealth adoption, such as ethnicity, migration background, and primary language, were not collected. This restricts our ability to assess disparities along these dimensions and may obscure differential patterns of digital engagement. Finally, income was measured with a coarse, self-developed scale that bunched many participants into the highest category, limiting socioeconomic resolution. A more granular income measure would have provided a clearer picture of the socioeconomic background of app users.

### Conclusion

In summary, users of the hypertension self-management app in Germany were predominantly well-educated, digitally literate individuals with a history of hypertension. While the app was rated as useful and showed high engagement, particularly for tracking health data and preparing for medical appointments, its formal integration into clinical care remained limited. Furthermore, user profiles point to a persistent digital divide, suggesting that socioeconomically disadvantaged groups are underrepresented. Simply making an app available is insufficient; who uses it, how it is used, and whether it is integrated into care are critical. Future research should test strategies to engage underrepresented groups, strengthen confidence in digital health information, and link patient-facing apps more effectively with clinical care so that mHealth can be more inclusive and effective in managing hypertension.

## Supplementary material

10.2196/83075 Multimedia Appendix 1 German questionnaire.

10.2196/83075 Multimedia Appendix 2 English version of the questionnaire.

10.2196/83075 Multimedia Appendix 3 Association between app usage frequency and sociodemographic variables.[Aff aff1]

## Abbreviations

eHEALS: eHealth Literacy Scale
mHealth: mobile health
SBP: systolic blood pressure
SUS: system usability scale
