# Correction: User Profiles and Engagement in a Hypertension Self-Management App: Cross-Sectional Survey

**DOI:** 10.2196/93515

**Published:** 2026-02-24

**Authors:** Felix Muehlensiepen, Susann May, Frances Seifert, Eileen Wengemuth, Olen Johannsen, Martin Middeke, Martin Heinze, Pascal Petit, Nicolas Vuillerme, Sebastian Spethmann, Dunja Bruch

**Affiliations:** 1 Center for Health Services Research, Faculty of Health Sciences Brandenburg, Brandenburg Medical School, Rüdersdorf, Seebad 82/83, Ruedersdorf bei Berlin, 15562, Germany, 49 01636055860; 2 Department of Cardiology, Angiology and Intensive Care Medicine, Deutsches Herzzentrum der Charité, Berlin, Germany; 3 Charité – Universitätsmedizin Berlin, corporate member of Freie Universität Berlin and Humboldt-Universität zu Berlin, Berlin, Germany; 4 Université Grenoble Alpes, CNRS, Grenoble INP, LIG Sangria, Grenoble, France; 5 Hypertension Care UG, Ingolstadt, Germany; 6 Hypertension Center Munich, Munich, Germany; 7 Center for Mental Health, Brandenburg Medical School, Immanuel Klinik Rüdersdorf, Ruedersdorf bei Berlin, Germany; 8 Department of Cardiovascular Surgery, Heart Center Brandenburg, Faculty of Health Sciences, Brandenburg Medical School Theodor Fontane, Bernau, Germany

In “User Profiles and Engagement in a Hypertension Self-Management App: Cross-Sectional Survey” [1], the authors noted an error in the order of affiliations 1 to 6, which was introduced during the production process.

The affiliation order was revised from the following:

^1^Hypertension Center Munich, Munich, Germany

^2^Center for Health Services Research, Faculty of Health Sciences Brandenburg, Brandenburg Medical School, Rüdersdorf, Ruedersdorf bei Berlin, Germany

^3^Department of Cardiology, Angiology and Intensive Care Medicine, Deutsches Herzzentrum der Charité, Berlin, Germany

^4^Charité – Universitätsmedizin Berlin, corporate member of Freie Universität Berlin and Humboldt-Universität zu Berlin, Berlin, Germany

^5^Université Grenoble Alpes, CNRS, Grenoble INP, LIG Sangria, Grenoble, France

^6^Hypertension Care UG, Ingolstadt, Germany

The order of the affiliations now reads:

^1^Center for Health Services Research, Faculty of Health Sciences Brandenburg, Brandenburg Medical School, Rüdersdorf, Ruedersdorf bei Berlin, Germany

^2^Department of Cardiology, Angiology and Intensive Care Medicine, Deutsches Herzzentrum der Charité, Berlin, Germany

^3^Charité – Universitätsmedizin Berlin, corporate member of Freie Universität Berlin and Humboldt-Universität zu Berlin, Berlin, Germany

^4^Université Grenoble Alpes, CNRS, Grenoble INP, LIG Sangria, Grenoble, France

^5^Hypertension Care UG, Ingolstadt, Germany

^6^Hypertension Center Munich, Munich, Germany

The correction will appear in the online version of the paper on the JMIR Publications website, together with the publication of this correction notice. Because this was made after submission to PubMed, PubMed Central, and other full-text repositories, the corrected article has also been resubmitted to those repositories.
