# Supplementary material for: User Profiles and Engagement in a Hypertension Self-Management App: Cross-Sectional Survey
Source: J Med Internet Res. 2026 Feb 11;28:e83075. doi: 10.2196/83075 (PMC12893642; doi:10.2196/83075)
Supplement: Multimedia Appendix 1 [file jmir-v28-e83075-s001.pdf]

MEDIZINISCHE HOCHSCHULE BRANDENBURG

Fragebogen zur Hypertonie.App

Herzlich Willkommen!

Vielen Dank, dass Sie an unserer Studie teilnehmen.
Bitte beantworten Sie die folgenden Fragen.
Wählen Sie jeweils die Antwortmöglichkeit aus, die am besten für Sie passt.

1. App-Nutzung

1.1 Seit wann nutzen Sie die App?

O Seit weniger als einem Monat
O Seit 1 - 6 Monaten
O Seit 6 - 12 Monaten
O Seit mehr als einem Jahr
O Ich nutze die App nicht mehr.

1.2 Wie häufig nutzen Sie die App?

O Täglich
O Mindestens einmal pro Woche
O Mindestens einmal pro Monat
O Seltener als einmal pro Monat
O Ich habe die App früher genutzt, aktuell nicht mehr.
O Ich habe die App noch nie benutzt.
→ Wenn Sie die App noch nie genutzt haben, können Sie nicht an dieser Befragung teilnehmen. Vielen Dank für Ihr Interesse!

1.3 Wofür nutzen Sie die App?

Mehrfachauswahl möglich

☐ Ich **dokumentiere** meine Blutdruckwerte.
☐ Ich erstelle **Berichte** für meine Arztbesuche.
☐ Ich nutze die **Tagebuchfunktion** und trage z.B. Symptome, Stress oder Gewicht ein.
☐ Ich lese die für mich **vorgeschlagenen Gesundheitsinformationen** ("Ratgeber").
☐ Ich lese die **allgemeinen Gesundheitsinformationen** ("gesamte Bibliothek").
☐ Ich nutze die **Erinnerungsfunktion**, z.B. für meine Medikamente oder Blutdruckmessung.
☐ Ich nutze die Anleitung zur **Tiefenatmung**.
☐ Andere Art der Nutzung, und zwar:

____

1.4 Wurde bzw. wird die App in Ihre **ärztliche Behandlung** einbezogen? Wenn ja, inwiefern?

Mehrauswahl möglich

☐ Ja, ich nutze meine **Blutdruck-Eintragungen** für den Arztbesuch.
☐ Ja, mein Arzt/ meine Ärztin passt meine **Medikamente** meinen Eintragungen entsprechend an.
☐ Ja, mein Arzt/ meine Ärztin hat mir die **Funktionen der App** erklärt.
☐ Ja, wir tauschen uns über die **Ratgeber-Informationen** aus.
☐ Nein, die App wird **nicht einbezogen**.
☐ Noch nicht, aber **ich habe es vor**.
☐ Andere Art der Einbeziehung, und zwar:

____

1.5 Wie sind Sie auf die App aufmerksam geworden?

Mehrfachauswahl möglich

☐ Empfehlung meiner Ärztin/ meines Arztes
☐ Empfehlung aus meinem privaten Umfeld
☐ Anzeige in Print-Medien
☐ Online-Anzeige
☐ Ich habe selbst im App-Store/ im Internet nach Blutdruck-Apps gesucht.
☐ Auf anderem Weg, und zwar:

____

1.6 Was war der ausschlaggebende Grund dafür, dass Sie angefangen haben, die App zu nutzen?

Mehrfachauswahl möglich

☐ Ich war **neugierig**.
☐ Die App ist mir durch meine Ärztin/ meinen Arzt **empfohlen** worden.
☐ Ich wollte etwas für meine **Gesundheit** tun.
☐ Ich wollte meine **Blutdruckwerte dokumentieren**.
☐ Ich wollte mir **Informationen** zu Bluthochdruck einholen.
☐ Ich wollte die **Tiefenatmung** ausprobieren.
☐ Anderer Grund, und zwar:

____

1.7 Welche der Funktionen finden Sie am hilfreichsten?
Mehrfachauswahl möglich

- ☐ **Dokumentieren** meiner Blutdruckwerte
- ☐ Erstellung von **Berichten** für meinen Arztbesuch
- ☐ Andere **Tagebucheinträge**, z.B. zu Symptomen, Stress oder Gewicht
- ☐ **Direktes Feedback** zu meinen Blutdruckwerten
- ☐ Für mich **angepasste Gesundheitsinformationen** ("Ratgeber")
- ☐ **Allgemeine Gesundheitsinformationen** ("gesamte Bibliothek")
- ☐ **Erinnerungsfunktion**, z.B. für Medikamenteneinnahme oder Blutdruckmessung
- ☐ Anleitung für die **Tiefenatmung**
- ☐ Verbindung zu **Apple Health/ Google Fit**
- ☐ Andere Funktion, und zwar:

____

1.8 Im Folgenden geht es um die Hypertonie.App und Ihre persönliche Einschätzung. Bitte beurteilen Sie, inwieweit Sie den Aussagen zustimmen.

	Stimme über-haupt nicht zu	Stimme nicht zu	Weder noch	Stimme zu	Stimme voll und ganz zu
Ich denke, ich würde die App regelmäßig (weiter) nutzen.	O	O	O	O	O
Die App erscheint mir unnötig kompliziert.	O	O	O	O	O
Ich finde, die App ist einfach zu benutzen.	O	O	O	O	O
Ich denke, ich bräuchte technische Unterstützung, um die App nutzen zu können.	O	O	O	O	O
Ich finde, dass die verschiedenen Funktionen der App gut integriert sind.	O	O	O	O	O
Ich finde, dass es in der App zu viele Unstimmigkeiten gibt.	O	O	O	O	O
Ich glaube, dass die meisten Leute die Benutzung der App schnell erlernen können.	O	O	O	O	O
Die App erscheint mir sehr umständlich zu bedienen.	O	O	O	O	O
Ich fühle mich bei der Nutzung der App sehr sicher.	O	O	O	O	O
Ich musste einiges lernen, um mit der App zurecht zu kommen.	O	O	O	O	O

2 Gesundheit

2.1 Wie sind Ihre aktuellen Blutdruckwerte ungefähr im Mittel?

Möglichst Selbstmessung im Ruhezustand

Systolisch (mmHG, „obere Wert“)	Diastolisch (mmHG, „unterer Wert“)
O < 120	O < 80
O 120-129	O 80-84
O 130-139	O 85-89
O 140-159	O 90-99
O 160-179	O 100-109
O ≥ 180	O ≥ 110
O Weiß nicht	O Weiß nicht

2.2 Nehmen Sie aktuell blutdrucksenkende Medikamente?

O Ja
O Nein
O Weiß nicht

2.3 Wann wurde bei Ihnen erstmalig Bluthochdruck diagnostiziert?

O Vor weniger als einem Jahr
O Vor 1 - 5 Jahren
O Vor 5 - 10 Jahren
O Vor mehr als 10 Jahren
O Ich hatte nie Bluthochdruck.
O Ich hatte früher Bluthochdruck und nun nicht mehr.

2.4 Welche anderen Erkrankungen haben/ hatten Sie neben Ihrem Bluthochdruck?

Mehrfachauswahl oder keine Auswahl möglich

☐ Herzinfarkt oder Schlaganfall
☐ Durchblutungsstörung der Herzkranzgefäße oder der Beinarterien (Schaufensterkrankheit)
☐ Herz- oder Gefäßoperationen
☐ Diabetes mellitus
☐ Erhöhte Cholesterinwerte oder Einnahme von cholesterinsenkenden Medikamenten
☐ Chronische Nierenerkrankung
☐ Andere chronische oder schwere Erkrankungen:

____

2.5 Rauchen Sie?
O Ja, ich rauche.
O Nein, aber ich habe früher regelmäßig geraucht.
O Nein, ich habe nie regelmäßig geraucht.

2.6 Bestehen bei Ihnen körperliche Einschränkungen, die eine regelmäßige körperliche Aktivität verhindern?
O Ja
O Nein

Falls Nein: Haben Sie in der letzten Zeit körperliche Aktivität (z.B. Gehen, Joggen, Radfahren oder Schwimmen) an 5 bis 7 Tagen pro Woche für mindestens 30 Minuten ausgeübt?
Bitte kreuzen Sie die Aussage an, die auf Sie am besten zutrifft.

Nein, und ich habe es auch nicht vor.	Nein, aber ich denke darüber nach.	Nein, aber ich habe die feste Absicht dazu.	Ja, aber es fällt mir sehr schwer.	Ja, und es fällt mir leicht.
O	O	O	O	O

2.7 Im Folgenden geht es um gesundheitsbezogene Informationen aus dem Internet und um Ihre persönliche Einschätzung.
Bitte kreuzen Sie die für Sie zutreffende Aussage an.

	Stimme über-haupt nicht zu	Stimme nicht zu	Weder noch	Stimme zu	Stimme voll und ganz zu
Ich weiß, welche Quellen für Gesundheitsinformationen im Internet verfügbar sind.	O	O	O	O	O
Ich weiß, **wo** ich im Internet nützliche Gesundheitsinformationen finden kann.	O	O	O	O	O
Ich weiß, **wie** ich im Internet nützliche Gesundheitsinformationen finde.	O	O	O	O	O
Ich weiß, wie ich das Internet nutzen kann, um Antworten auf meine Fragen rund um das Thema Gesundheit zu bekommen.	O	O	O	O	O
Ich weiß, wie ich Informationen aus dem Internet so nutzen kann, dass sie mir weiterhelfen.	O	O	O	O	O

	Stimme überhaupt nicht zu	Stimme nicht zu	Weder noch	Stimme zu	Stimme voll und ganz zu
Ich bin in der Lage, Informationen, die ich im Internet finde, kritisch zu bewerten.	O	O	O	O	O
Ich kann im Internet zuverlässige von fragwürdigen Informationen unterscheiden.	O	O	O	O	O
Wenn ich gesundheitsbezogene Entscheidungen auf Basis von Informationen aus dem Internet treffe, fühle ich mich dabei sicher.	O	O	O	O	O

3. Mobilität und Verfügbarkeit

3.1 Bitte geben Sie die ungefähre Fahrtzeit bis zu Ihrem Arzt/ Ihrer Ärztin, der/ die Ihren Bluthochdruck behandelt, an.

______ Minuten

3.2 Welche der folgenden Verkehrsmittel stehen Ihnen zur Verfügung, um zu Ihrem Arzt/ Ihrer Ärztin zu kommen?
Mehrfachauswahl möglich

- ☐ Auto
- ☐ Fahrrad/ Elektrorad
- ☐ Bus/ Bahn
- ☐ Nichts davon
- ☐ Andere, und zwar: ________________________

3.3 Wie ist die Qualität und Geschwindigkeit Ihrer Internetverbindung?
Denken Sie bitte an die Ladegeschwindigkeit von Internetseiten, das Schauen von Videos (z.B. bei YouTube, Netflix) oder an Online-Meetings (z.B. über Skype, zoom).

- O Keine Probleme.
- O Teilweise längere Ladezeiten oder Abbruch der Verbindung. Meist aber problemlos.
- O Oft lange Ladezeiten und häufige Abbrüche der Verbindung.
- O Ich weiß nicht.
- O Ich habe keinen Internetzugang.

3.4 Welche der folgenden technischen Kommunikationsgeräte besitzen Sie?
Mehrfachauswahl möglich

- ☐ Telefon
- ☐ Smartphone
- ☐ Computer/ Laptop
- ☐ Tablet
- ☐ Smartwatch/ Fitnesstracker
- ☐ Faxgerät
- ☐ Andere, und zwar: ________________________

4. Soziodemographische Merkmale

Im Folgenden stellen wir Ihnen einige Fragen zu Ihrer Person. Die Angaben helfen, die Ergebnisse dieser Umfrage auszuwerten und einzuordnen. Außerdem können wir so überprüfen, ob unsere Teilnehmenden repräsentativ für die Gesamtbevölkerung sind. Wir möchten Sie nochmals darauf hinweisen, dass die Befragung anonym ist.

4.1 Wie alt sind Sie?

______ Jahre

Welchem Geschlecht ordnen Sie sich zu?

- O männlich
- O weiblich
- O divers

4.2 Was ist Ihr höchster Bildungsabschluss?

- O Ich bin aktuell Schüler/-in.
- O Kein Schulabschluss
- O Hauptschulabschluss (Volksschulabschluss) oder gleichwertiger Abschluss
- O Realschulabschluss (Mittlere Reife), Abschluss polytechnische Oberschule oder gleichwertiger Abschluss
- O Abitur, fachgebundene Hochschulreife, Abschluss erweiterte Oberschule oder gleichwertiger Abschluss
- O Hochschulabschluss oder Fachhochschulabschluss
- O Anderer Abschluss: ____

4.3 Wie hoch ist Ihr durchschnittliches monatliches Netto-Einkommen?
Summe aus: Lohn/ Gehalt, selbstständige Tätigkeit, Rente/ Pension, öffentliche Beihilfen, Vermietung/ Verpachtung, sonstige Einkünfte minus Steuern und Sozialversicherungsbeiträge

O unter 1000 €
O 1000 - 1500 €
O 1500 - 2500 €
O mehr als 2500 €
O keine Angabe

4.4 Wie groß ist der Ort, in dem Sie wohnen?

O Ländliche Region (Gemeinde unter 5.000 Einwohner)
O Kleinstadt (5.000 – 20.000 Einwohner)
O Mittelstadt (20.000 – 100.000 Einwohner)
O Großstadt (über 100.000 Einwohner)
O In welchem Bundesland leben Sie überwiegend?

5. Sonstige Kommentare

____

____

____

____

____

Vielen Dank für Ihre Teilnahme an der Befragung!

Um den **15€ Gutschein** zu erhalten, füllen Sie bitte das gelbe Gutschein-Formular aus.
Sie können über das Formular auch angeben, wenn Sie an einem einmaligen und vertraulichen **Telefoninterview** im Rahmen der Studie interessiert sind (Dauer ca. 30min, Vergütung 30€).

Bei Fragen wenden Sie sich gerne an unser Studienteam:

Dunja Bruch
Studienleiterin
Medizinische Hochschule Brandenburg
Telefon: 03338 694543
Mobil: 0162 2388926
E-Mail: dipah@mhb-fontane.de
Webseite: https://www.mhb-fontane.de/dipah.html
