# Supplementary material for: User Profiles and Engagement in a Hypertension Self-Management App: Cross-Sectional Survey
Source: J Med Internet Res. 2026 Feb 11;28:e83075. doi: 10.2196/83075 (PMC12893642; doi:10.2196/83075)
Supplement: Multimedia Appendix 2 [file jmir-v28-e83075-s002.pdf]

MEDIZINISCHE HOCHSCHULE BRANDENBURG

Questionnaire about the Hypertension App

Welcome!

Thank you for participating in our study.
Please answer the following questions.
Select the answer option that best suits you in each case.

1. App-Use

1.1 Since when are you using this app?

O Less than a month
O For 1-6 months
O For 6 - 12 months
O For more than a year
O I am not using the app anymore

1.2 How often do you use the app?

O daily
O At least once a week
O At least once a month
O Less than once a month
O I used to use the app, but I don´t anymore
O I never used the app
→ If you´ve never used the app, you cannot participate in this study. Thank you for your interest!

1.3 What do you use the app for?
Multiple selections possible

☐ I **document** my blood pressure readings
☐ I create **reports** for my doctor´s appointments
☐ I use the **diary function** and enter symptoms, stress levels or weight, for example
☐ I read the **health information suggested** for me ('advice')
☐ I read the **general health information** ('entire library')
☐ I use the **reminder function**, e.g. for my medication of blood pressure measurement
☐ I use the **deep breathing** guide
☐ Other type of use, namely:

____

1.4 Has the app been or is it being incorporated into your medical treatment? If so, to what extent?
Multiple selections possible

- ☐ Yes, I use my **blood pressure readings** when I visit my doctor
- ☐ Yes, my doctor adjusts my **medication** based on my readings
- ☐ Yes, my doctor explained the **app´s features** to me
- ☐ Yes, we discuss the **information provided in the guide**
- ☐ No, the app is **not included**
- ☐ Not yet, but I **plan** to
- ☐ Other type of inclusion, namely:

____

1.5 How did you find out about the app?

Multiple selections possible

- ☐ Recommendation from my doctor
- ☐ Recommendation from my personal circle
- ☐ Advertisement in print media
- ☐ Online advertisement
- ☐ I searched for blood pressure apps myself in the app store/the internet
- ☐ Other means, namely:

____

1.6 What was the main reason you started using the app?

Multiple selections possible

- ☐ I was **curious**
- ☐ The app was **recommended** to me by my doctor
- ☐ I wanted to do something for my **health**
- ☐ I wanted to document my **blood pressure readings**
- ☐ I wanted to obtain **information** about high blood pressure
- ☐ I wanted to try **deep breathing**
- ☐ Other reason, namely:

____

1.7 Which oft the features do you find most helpful?

Multiple selections possible

- ☐ **Documenting** my blood pressure readings
- ☐ Creating **reports** for my doctor’s appointment
- ☐ Other **diary entries**, e.g. on symptoms, stress or weight
- ☐ **Direct feedback** on my blood pressure readings
- ☐ **Health information tailored** to me (‘advice’)
- ☐ **General** health information (‘entire library’)

- ☐ **Reminder function,** e.g. for taking medication or measuring blood pressure
- ☐ Instructions for **deep breathing**
- ☐ **Connection** to Apple Health/Google Fit
- ☐ Other function, namely:

____

1.8 The following section concerns the Hypertension App and your personal assessment. Please rate the extent to which you agree with the statements.

	I completely disagree	I disagree	Neither	I agree	I completely agree
I think I (continually) would use the app regularly.	O	O	O	O	O
The app seems unnecessarily complicated to me.	O	O	O	O	O
I find the app easy to use.	O	O	O	O	O
I think I would need technical support to be able to use the app.	O	O	O	O	O
I think that the various functions of the app are well integrated.	O	O	O	O	O
I think there are too many inconsistencies in the app.	O	O	O	O	O
I believe that most people can quickly learn how to use the app.	O	O	O	O	O
The app seems to be very awkward to use.	O	O	O	O	O
I feel very secure when using the app.	O	O	O	O	O
I had to learn quite a bit to get used to the app.	O	O	O	O	O

2 Health

2.1 What is your current average blood pressure reading?

If possible, measure your own blood pressure at rest.

Systolic (mmHg, ‘upper value’)	Diastolic (mmHg, ‘lower value’)
O < 120<br>O 120-129<br>O 130-139	O < 80<br>O 80-84<br>O 85-89

O 140-159	O 90-99
O 160-179	O 100-109
O ≥ 180	O ≥ 110
O I don´t know	O I don´t know

2.2 Are you currently taking blood pressure medication?
O Yes
O No
O I don´t know

2.3 When were you first diagnosed with high blood pressure?
O Less than a year ago
O 1 - 5 years ago
O 5 – 10 years ago
O More than 10 years ago
O I´ve never had high blood pressure
O I used to have high blood pressure, but I don´t anymore

2.4 What other illnesses do you have/have you had besides high blood pressure?
Multiple selections possible or no answer at all
□ Heart attack or stroke
□ Circulatory disorder of the coronary arteries or leg arteries (intermittent claudication)
□ Heart or vascular surgery
□ Herz- oder Gefäßoperationen
□ Diabetes mellitus
□ Elevated cholesterol levels or use of cholesterol-lowering medication
□ Chronic kidney disease
□ Other chronic or serious illnesses:

____

2.5 Do you smoke?
O Yes, I smoke.
O No, but I used to smoke regularly.
O No, I have never smoked regularly.

2.6 Do you have any physical limitations that prevent you from engaging in regular physical activity?
O Yes

O No

If no: Have you recently been doing physical activity (e.g. walking, jogging, cycling or swimming) for at least 30 minutes on 5 to 7 days a week?
Please select the statement that best applies to you.

No, and I don't intend to.	No, but I'm considering it.	No, but I fully intend to.	Yes, but I find it very difficult.	Yes, and it's easy for me.
O	O	O	O	O

2.7 The following section deals with health-related information from the Internet and your personal assessment.
Please rate the extent to which you agree with the statements.

	I completely disagree	I disagree	Neither	I agree	I completely agree
I know which sources of health information are available on the internet.	O	O	O	O	O
I know **where** to find useful health information on the Internet.	O	O	O	O	O
I know **how** to find useful health information on the internet.	O	O	O	O	O
I know how to use the internet to find answers to my questions about health.	O	O	O	O	O
I know how to use information from the internet in a way that helps me.	O	O	O	O	O
	I completely disagree	I disagree	Neither	I agree	I completely agree
I am able to critically evaluate information that I find on the internet.	O	O	O	O	O
I can differentiate between reliable and questionable information on the internet.	O	O	O	O	O
When I make health-related decisions based on information from the internet, I feel confident about them.	O	O	O	O	O

3. Mobility and availability

3.1 Please indicate the approximate travel time to your doctor who treats your high blood pressure.

______ Minuten

3.2 Which of the following transport options are available to you for travelling to your doctor's office?
Multiple selections possible

- ☐ Car
- ☐ Bicycle/electric bike
- ☐ Bus/train
- ☐ None of the above
- ☐ Other, namely: ________________

3.3 How is the quality and speed of your internet connection?
Please consider the loading speed of websites, watching videos (e.g. on YouTube, Netflix) or online meetings (e.g. via Skype, Zoom).

- O No problems
- O Occasional longer loading times or connection interruptions. But mostly no problems.
- O Often long loading times and frequent disconnections.
- O I don´t know.
- O I don't have internet access.

3.4 Which of the following technical communication devices do you own?
Multiple selections possible

- ☐ Telephone
- ☐ Smartphone
- ☐ Computer/ Laptop
- ☐ Tablet
- ☐ Smartwatch/ Fitness tracker
- ☐ Fax machine
- ☐ Others, namely _______________________

4. Sociodemographic characteristics

Below, we will ask you a few questions about yourself. The information you provide will help us to evaluate and classify the results of this survey. It will also enable us to check whether our participants are representative of the general population.

We would like to remind you once again that this survey is anonymous.

4.1 How old are you?

______ years

What gender do you identify as?

O male
O female
O diverse

4.2 What is your highest level of education?

O I am currently a student.
O No school leaving certificate.
O Basic secondary school qualification or equivalent qualification
O General Certificate of Secondary Education, polytechnic secondary school leaving certificate or equivalent qualification
O A-levels, subject-specific university entrance qualification, advanced secondary school qualification or equivalent qualification
O University degree or technical college degree
O Other qualification: ____

4.3 What is your average monthly net income?

Total of: wages/salary, self-employment, pension, public subsidies, rental income, other income minus taxes and social security contributions

O Less than 1000 €
O 1000 - 1500 €
O 1500 - 2500 €
O more than 2500 €
O no information

4.4 How big is the town you live in?

O Rural area (municipality with fewer than 5.000 inhabitants)
O Small town (5.000 – 20.000 inhabitants)
O Medium-sized town (20.000 – 100.000 inhabitants)
O Large town (more than 100.000 inhabitants)
O In which federal state do you mainly live? ____________________

5. Other comments

____

____

____

____

____

Thank you for participating in the survey!

To receive the **15€ voucher**, please fill out the **yellow** voucher form.
You can also use the form to indicate if you are interested in participating in a **one-time**, confidential **telephone interview** as part of the study
(duration approx. 30 minutes, compensation 30€).

If you have any questions, please contact our study team:

Dunja Bruch
Study Director
Medical School Brandenburg
Telephone: 03338 694543
Mobile: 0162 2388926
Email: dipah@mhb-fontane.de
Website: https://www.mhb-fontane.de/dipah.html
