# Supplementary material for: User Profiles and Engagement in a Hypertension Self-Management App: Cross-Sectional Survey
Source: J Med Internet Res. 2026 Feb 11;28:e83075. doi: 10.2196/83075 (PMC12893642; doi:10.2196/83075)
Supplement: Multimedia Appendix 3 [file jmir-v28-e83075-s003.pdf]

Association between app usage frequency and sociodemographic variables

| Variable | Category | OR [95% CI] | t value | p value | adjusted p value |
|---|---|---|---|---|---|
| Systolic blood pressure | <120 mmHG | 1 (reference) | | | |
| Systolic blood pressure | 120-129 mmHG | 0.70 [0.32-1.52] | -0.90 | 0.37 | 0.57 |
| Systolic blood pressure | 130-139 mmHG | 0.54 [0.26-1.15] | -1.59 | 0.11 | 0.32 |
| Systolic blood pressure | **140-159 mmHG** | **0.34 [0.15-0.76]** | **-2.63** | **8.45e-03** | **0.05** |
| Systolic blood pressure | **160-179 mmHG** | **0.09 [0.02-0.51]** | **-2.73** | **6.32e-03** | **0.04** |
| Diastolic blood pressure | <80 mmHG | 1 (reference) | | | |
| Diastolic blood pressure | 80-84 mmHG | 5.57 [0.55-56.8] | 1.45 | 0.15 | 0.35 |
| Diastolic blood pressure | 85-89 mmHG | 5.75 [0.56-59.3] | 1.47 | 0.14 | 0.35 |
| Diastolic blood pressure | 90-99 mmHG | 4.28 [0.42-43.8] | 1.22 | 0.22 | 0.47 |
| Diastolic blood pressure | 100-109 mmHG | 2.73 [0.27-28.1] | 0.84 | 0.40 | 0.59 |
| Diastolic blood pressure | > 110 mmHG | 2.11 [0.14-31.0] | 0.54 | 0.59 | 0.74 |
| Time of initial diagnosis | less than a year ago | 1 (reference) | | | |
| **Time of initial diagnosis** | **5 - 10 years ago** | **4.91 [2.06-11.7]** | **3.58** | **3.43e-04** | **3.88e-03** |
| **Time of initial diagnosis** | **more than 10 years ago** | **3.29 [1.63-6.64]** | **3.32** | **8.95e-04** | **7.61e-03** |
| **Time of initial diagnosis** | **15 years ago** | **6.78 [3.54-13.1]** | **5.75** | **8.91e-09** | **3.03e-07** |
| Travel time to doctor | | 1.00 [0.99-1.02] | 0.69 | 0.49 | 0.69 |
| Age (years) | | 1.00 [0.99-1.03] | 1.01 | 0.31 | 0.53 |
| Monthly income | Earning less than 1000 euros per month | 1 (reference) | | | |
| Monthly income | Earning 1000-1500 euros per month | 0.77 [0.23-2.59] | -0.42 | 0.67 | 0.79 |
| Monthly income | Earning 1500-2500 euros per month | 0.81 [0.28-2.38] | -0.38 | 0.70 | 0.80 |
| Monthly income | Earning more than 2500 euros per month | 1.79 [0.65-4.90] | 1.13 | 0.26 | 0.49 |
| City of residence | Living in a rural region (municipality with less than 5,000 inhabitants) | 1 (reference) | | | |
| **City of residence** | **Living in a small town (5,000-20,000 inhabitants)** | **2.08 [0.99-4.38]** | **1.94** | **0.05** | **0.19** |
| City of residence | Living in a medium-sized town (20,000-100,000 inhabitants) | 1.63 [0.72-3.69] | 1.18 | 0.24 | 0.48 |
| City of residence | Living in a large city (over 100,000 inhabitants) | 1.66 [0.83-3.33] | 1.42 | 0.16 | 0.35 |
| Gender | Female | 0.92 [0.59-1.45] | -0.35 | 0.73 | 0.80 |
| Gender | Male | 1.12 [0.72-1.76] | 0.51 | 0.61 | 0.74 |
| Smoking status | Being a smoker | 0.81 [0.38-1.75] | -0.53 | 0.59 | 0.74 |
| Smoking status | Not smoking but has been a regular smoker | 0.86 [0.53-1.38] | -0.63 | 0.53 | 0.72 |
| Smoking status | Not smoking and never smoked regularly | 1.23 [0.78-1.94] | 0.91 | 0.36 | 0.57 |
| Education level | Being a student | 5.71e-07 [0-Inf] | -0.02 | 0.98 | 0.98 |
| Education level | Having no degree | 1.17 [0.06-21.5] | 0.11 | 0.92 | 0.94 |
| Education level | Having a secondary school leaving certificate or equivalent qualification | 0.53 [0.25-1.12] | -1.66 | 0.10 | 0.30 |
| Education level | **Having a secondary school diploma, polytechnic high school diploma or equivalent qualification** | **0.49 [0.27-0.89]** | **-2.33** | **0.02** | **0.10** |
| Education level | **Having a high school diploma, subject-specific university entrance qualification, advanced high school diploma or equivalent qualification** | **0.58 [0.34-1.01]** | **-1.92** | **0.05** | **0.19** |
| Education level | **Having a university degree or technical college degree** | **2.58 [1.63-4.08]** | **4.05** | **5.06e-05** | **8.60e-04** |
| Education level | **Having another degree** | **4.70 [1.01-22.0]** | **1.96** | **0.05** | **0.19** |
